# Unique genetic background and outcome of non‐Caucasian Japanese probands with arrhythmogenic right ventricular dysplasia/cardiomyopathy

**DOI:** 10.1002/mgg3.311

**Published:** 2017-08-13

**Authors:** Yuko Wada, Seiko Ohno, Takeshi Aiba, Minoru Horie

**Affiliations:** ^1^ Department of Cardiovascular Medicine Shiga University of Medical Science Otsu Japan; ^2^ Department of Cardiovascular Medicine National Cerebral and Cardiovascular Center Suita Japan

**Keywords:** Arrhythmogenic right ventricular dysplasia/cardiomyopathy, genetics, phenotype, prognosis, racial difference

## Abstract

**Background:**

Arrhythmogenic right ventricular dysplasia/cardiomyopathy (ARVD/C) is an inherited cardiomyopathy mainly caused by desmosomal gene mutation. More than half of Caucasian probands have desmosomal mutations, which lead to earlier onset of ventricular arrhythmias. Among non‐Caucasians, the genetic background of ARVD/C probands and its prognostic impact remain unclear.

**Methods and Results:**

We genotyped 99 unrelated Japanese ARVD/C probands for plakophilin 2 (*PKP2*), desmoglein 2 (*DSG2*), desmoplakin (*DSP*), and desmocollin 2 (*DSC2*) between 2005 and 2014. Seventy‐five probands who fulfilled “definite” category according to the 2010 Task Force Criteria (TFC) were enrolled and followed up for 6.4 years. Sixty‐four percent of probands had desmosomal mutations; *DSG2* was predominant (48% of mutations) followed by *PKP2* (38%). *DSG2* mutations were almost missense, whereas over 90% of *PKP2* mutations were truncating mutations. Lethal ventricular arrhythmias (VAs, sustained ventricular tachycardia/fibrillation) occurred in 57% of probands as the first manifestation and 71% at the end of follow‐up. Five died during follow‐up. Truncating mutation carriers exhibited earlier lethal VAs onset compared to missense mutation carriers or mutation negatives (age at onset 35 ± 12, 49 ± 16, and 50 ± 19 years, respectively, *P* < 0.05 in each). Cox proportional hazard analysis revealed for the first time that, compared to mutation negatives, truncating mutation carriers had higher risk for lethal VAs, and especially for onset by their 40s, in an age‐dependent manner (RR = 4.6, *P* < 0.01 by their 40s; RR = 2.9, *P* = 0.01 by their 50s).

**Conclusion:**

The genetic background of Japanese ARVD/C probands is distinct from that of Caucasian probands, leading to distinct prognosis. The most affected gene mutations in Japanese probands were missense mutations in *DSG2* leading to modest outcome, whereas *PKP2* truncating mutations were the second most and might be a strong marker for lethal VAs in non‐Caucasian Japanese ARVD/C probands.

## Introduction

Arrhythmogenic right ventricular dysplasia/cardiomyopathy (ARVD/C) is an inherited cardiomyopathy characterized by right ventricular structural impairment and electrophysiological disturbance, both attributed histologically to cellular loss and succeeding fibro‐fatty displacement of myocytes (Thiene 2015). ARVD/C is progressive after its onset, which can occur as early as the teen years, though molecular degradation can begin even earlier in life (Pilichou et al. 2009). Desmosomal gene mutations play a key role as triggers of disease development (Sen‐Chowdhry et al. 2005; Groeneweg et al. 2015) and have been identified in over half of Caucasian probands (Basso et al. 2012). Plakophilin 2 (*PKP2,*OMIM reference number*602861) mutations are significantly predominant in Caucasian (Groeneweg et al. 2015) and some Chinese probands (Bao et al. 2013; Zhou et al. 2015). In the previous report from our group, *PKP2* mutations were the most common among 29 probands with definite ARVD/C phenotype in a preliminary genetic study for Japanese (Ohno et al. 2013), although mutations were to be more sharply distinguished from variants. Regarding genetic background of non‐Caucasian ARVD/C patients and family members, its associated clinical phenotypes and outcomes have not yet been fully defined. In this study, we sought to examine the genetic background, phenotype and phenotype variation, and outcomes of mono‐racial non‐Caucasian Japanese ARVD/C probands over the long term.

## Methods

### Study population

This study was conducted as part of a comprehensive nationwide survey of inherited arrhythmias in Japan. Ninety‐nine unrelated Japanese probands suspected to have ARVD/C and 74 of their family members were genotyped between 2005 and 2014. Of the 99 consecutive probands, 75 who fulfilled the “definite” category according to the 2010 Task Force Criteria (TFC) (Marcus et al. 2010) were enrolled. Briefly, “definite” category was determined if proband sufficed any of two major, one major plus two minor, or four minor criteria in different phenotypic criteria as follows; “right ventricular structural alterations,” “tissue characterization in cardiac biopsy sample,” “repolarization abnormalities in electrocardiogram (ECG),” “depolarization abnormalities in ECG,” “arrhythmias,” and “family history” as commonly applied.

Of the 74 family members, 58 were enrolled whose proband relatives had met the 2010 TFC. All probands and family members gave written informed consent for both genetic testing and the research survey prior to genotyping. This study protocol was approved by the ethics committee of Shiga University of Medical Science (25‐167).

### Phenotype assessment

Each proband's ARVD/C diagnosis was reviewed by two cardiologists at different time points. First, one cardiologist examined each index proband and his or her family members, recording medical information including ECG, signal‐averaged electrocardiogram, echocardiogram, cardiac magnetic resonance imaging or right ventriculography, and Holter recording (“first evaluation”). The proband was then referred to the genetic laboratory. At this point a second cardiologist reviewed the original records and survey forms regarding the clinical diagnosis. Genotyping was performed and the proband was enrolled in the study (“genotyping and study enroll”). Finally, the initial cardiologist followed up with all probands and conducted a follow‐up survey (“final evaluation”).

To determine genotype–phenotype correlations, the odds for mutation‐positive status were derived from logistic analysis, performed as previously described (Te Riele et al. 2016), of all probands with “possible” TFC phenotype (i.e., with one major or two minor criteria in different phenotypic criteria as described above) or a more severe phenotype. We employed “the modified TFC” which are mutation‐independent and affected only by clinical phenotype. Probands with “possible” phenotype according to the 2010 TFC are supposed to be upgraded into the “definite” category by the presence of a pathogenic mutation, as this constitutes one additional major criterion. In the genotype–phenotype analysis, we employed the following indices in addition to the 2010 TFC: right ventricular fragmentation as observed in the form of fragmented QRS in V1‐3 leads in ECG was defined as depolarization anomaly; reduced left ventricular ejection fraction (LVEF) <50% as left ventricular dysfunction; J wave in two or more serial leads in either the anterior, inferior, or lateral leads in ECG. In phenotype progression analysis, each phenotype was quantified as follows: 2 points for a major anomaly, 1 point for a minor anomaly. A “definite” phenotype was defined as a score of 4 or more points, “borderline” as a 3‐point score, and “possible” as a 2‐point score.

### Genetic analysis

Genomic deoxyribonucleic acid (DNA) was isolated from venous blood lymphocytes as previously described (Ohno et al. 2013). Genotyping was performed according to a bidirectional direct DNA sequencing method targeting a comprehensive open reading frame/splice site mutational analysis of four major ARVD/C susceptibility genes: *PKP2* (OMIM reference number*602861), encoding plakophilin 2; *DSP* (*125647)*,* encoding desmoplakin; *DSG2* (*125671), encoding desmoglein 2; and *DSC2* (*125645), encoding desmocollin 2. To screen for nondesmosomal genes, all probands were genotyped for *LMNA* (*150330) and *SCN5A* (*600163), encoding lamin A/C and voltage gated sodium channel alpha subunit type V, respectively. Two probands carrying the *LMNA* mutation and their four relatives, all previously reported by our laboratory (Kato et al. 2016), and three probands carrying the *SCN5A* mutation either alone or in combination with desmosomal mutations and their eight relatives were excluded from this study. The coding DNA sequences of *PKP2*,* DSP*,* DSG2*,* DSC2, LMNA,* and *SCN5A* were based on the GenBank reference sequences; NM_004572.3, NM_001008844.2, NM_001943.4, NM_004949.4, NM_001257374.2, and NM_00335.4, respectively. Genetic screening of family members was focused on the anomalous gene(s) found in their respective probands.

To determine whether a repetitively identified variant p.Asp494Ala in *DSG2* is a recurrent or has a common founder, both repeat numbers of a microsatellite 15xGT located at ch18:31499358‐87 was analyzed using primers 5′‐GAGATTGTGCCACTGCACTC‐3′ and 5′‐CGACCACGGTAGGAATTCTG‐3′.

### Variant interpretation

Variants were heterozygotes if not specified as homozygotes. All null variants including nonsense, frameshift, canonical ±1 or 2 splice sites, or single/multiple exon deletion/duplication were considered pathogenic. Interpretation for missense variants is detailed in Appendix S1. Increased odds ratio (>5.0 as compared to Japanese controls) was considered as sufficient evidence of pathogenicity if there was a discrepancy in minor allele frequency (MAF) between probands and controls according to the latest guidelines for the interpretation of the variants (Richards et al. 2015). In that context, the variant p.Asp494Ala in *DSG2* (*DSG2*‐D494A) is a single exception that has been identified in the higher odds ratio (=26.9) for definitive ARVD/C. On the basis of its slightly higher MAF (0.006) in Japanese controls and the lack of convincing data that demonstrates its pathogenicity, we regarded this common variant as the variant of uncertain significance when identified as heterozygote. Homozygous mutation was regarded as pathogenic unless the available databases listed the index variant as a homozygote. For further analyses, pathogenic mutations were subclassified as either truncating mutation synonymous with the null variant or nontruncating mutation which indicated missense mutation. Multiple missense mutations included compound heterozygotes (trans‐), monogenic double mutations (cis‐), and homozygous mutations; the number of mutations harbored in each proband was also analyzed for comparison to previous report (Rigato et al. 2013).

### Follow‐up, outcome, and reevaluation

All probands and family members received a follow‐up by the initial cardiologist after genotyping. The phenotype and clinical course as of the date of that follow‐up were reported in detail to the second cardiologist (Genetic Testing Laboratory, Shiga University of Medical Science) via a survey form. The most recent survey forms were collected in September 2015. The endpoints thus identified were spontaneous lethal ventricular arrhythmias (VAs) including sudden cardiac death (SCD), resuscitated cardiopulmonary arrest (CPA), sustained ventricular tachycardia (VT), ventricular fibrillation (VF), and appropriate cardioversion by implantable cardioverter defibrillator (ICD) or cardiac resynchronization therapy with defibrillator (CRT‐D). Antitachycardia pacing therapy by ICD or CRT‐D was not included among the lethal events because it was sometimes set to fire against nonsustained VT.

### Statistics

Continuous variables were presented as means ± SD. Comparison between two or three groups was made by Student's *t*‐test or the Kruskal–Wallis test, respectively. Categorical variables were expressed as frequency (%) with absolute numbers and compared by the chi‐square test or the Mann–Whitney *U* test between two or three groups, respectively. For outcome analyses, patients were divided into groups based on mutation status. Kaplan–Meier survival curves were constructed by plotting age at onset. A two‐tailed probability (*P*) value <0.05 was accepted as significant. A software package (JMP 9.0; SAS Institute Inc., Cary, NC, USA) was used for statistical analysis.

## Results

### Phenotype of Japanese ARVD/C probands

**Figure 1:**
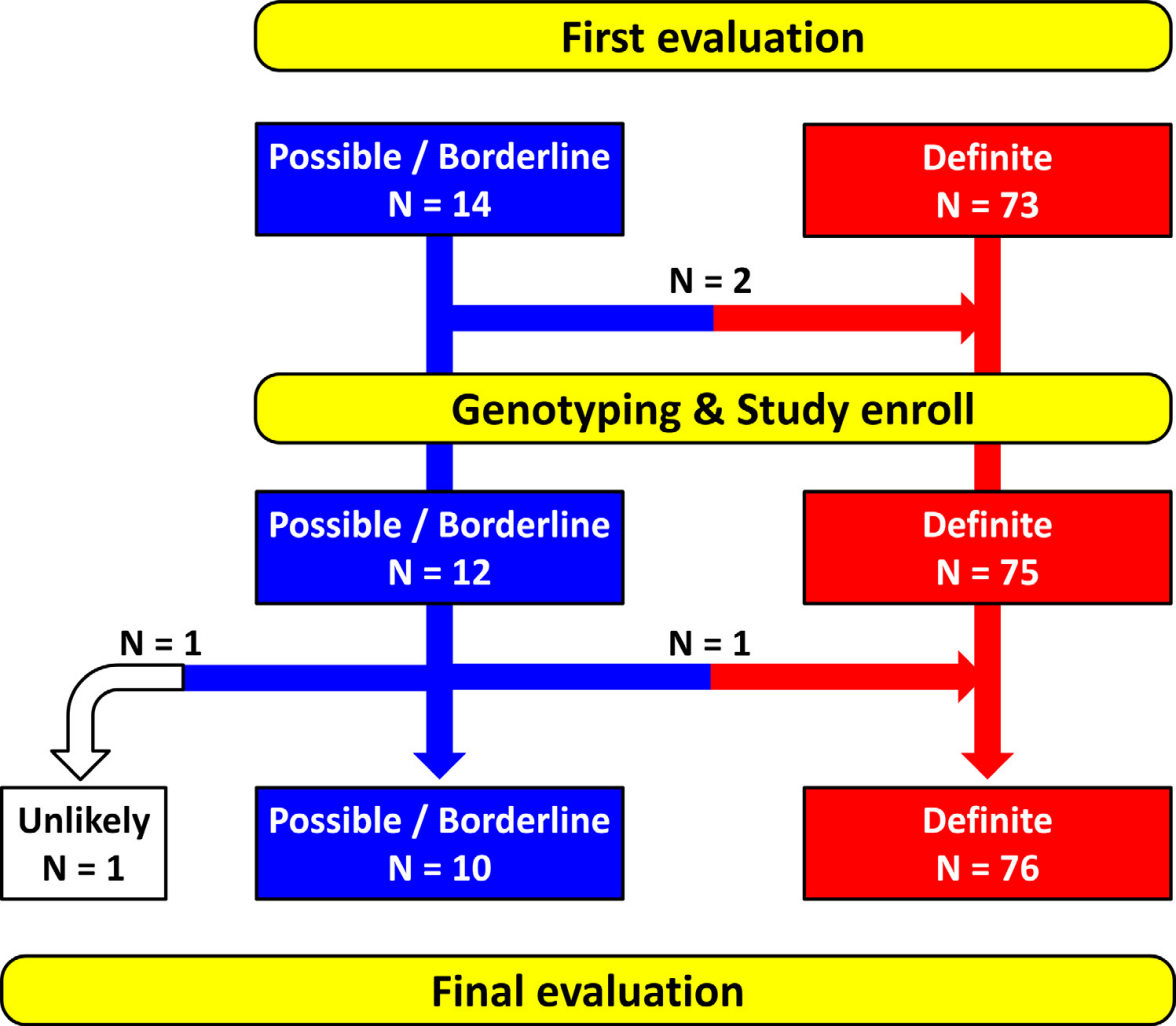
Time course of diagnoses and phenotype alteration during follow‐up. Note that diagnoses rarely changed during the mean follow‐up period of 6.4 years. “Definite,” “borderline,” and “possible” are defined as by the 2010 TFC. “Unlikely” means that the subject did not meet enough of the criteria to be sorted into the “possible” category.

Clinical diagnoses at the time of genotyping and disease progression are illustrated in Figure 1. Phenotype progressed during the study period in three probands; this occurred prior to genotyping in two probands and after genotyping in another one. The baseline clinical characteristics of the 75 probands at genotyping are shown in Table 1. The most common initial manifestation was lethal VAs (43 probands, 57%) followed by nonlethal VAs (12, 16%) and syncope (9, 12%). Of the 43 probands with previous lethal VAs before genotyping, three had been successfully rescued from CPA, whereas the remaining 40 had sustained VT as the initial manifestation. Prior hospitalization for heart failure was less common as the first symptom in this cohort. ICDs were implanted in total of 41 probands, 33 before and eight after genotyping. Family history of SCD in a first‐degree relative was reported by four of 75 probands; the same condition in a second‐degree relative was reported by seven.

**Table 1:** Clinical characteristics of 75 probands who fulfilled the 2010 Task Force Criteria

	Total *N* = 75	Mutation (+) *N* = 48	Mutation (−) *N* = 27	*P* value
Age at first evaluation, yrs	44 ± 18	42 ± 18	49 ± 18	0.08
Age at genotyping, yrs	49 ± 18	47 ± 18	52 ± 18	0.17
Age at final evaluation, yrs	50 ± 18	49 ± 18	54 ± 19	0.24
Follow‐up period, yrs	6.4 ± 6.2	7.2 ± 6.7	4.6 ± 4.8	0.08
Male, *n* (%)	55 (73)	35 (71)	20 (77)	0.61
SCD in 1st degree relative	4 (5)	3 (6)	1 (4)	0.66
SCD in 2nd degree relative	7 (9)	5 (10)	2 (7)	0.70
First manifestation				
Lethal VAs, *n* (%)	43 (57)	29 (60)	14 (52)	0.67
Nonlethal VAs, *n* (%)	12 (16)	7 (15)	5 (19)	0.58
Heart failure, *n* (%)	5 (7)	4 (8)	1 (4)	0.48
Family history of SCD, *n* (%)	2 (3)	2 (4)	0	0.30
Syncope, *n* (%)	9 (12)	5 (10)	4 (15)	0.51
Others, (%)	3 (4)	1 (2)	2 (8)	0.23
Major structural anomaly, (%)	51 (68)	32 (65)	19 (70)	0.49
Major repolarization anomaly, *n* (%)	47 (63)	35 (73)	12 (44)	0.03
Major depolarization anomaly, *n* (%)	29 (39)	17 (35)	12 (44)	0.33
Major ARVD/C‐related VAs, *n* (%)	18 (24)	10 (21)	8 (30)	0.31
Phenotype score at genotyping, pts	5.2 ± 1.7	5.2 ± 1.6	5.2 ± 1.8	0.65
LVDD, mm	47 ± 7	48 ± 7	47 ± 6	0.59
LVDS, mm	33 ± 7	33 ± 8	33 ± 5	0.82
LVEF, %	53 ± 15	51 ± 16	55 ± 12	0.41
ICD implantation at final evaluation, *n* (%)	41 (55)	24 (50)	10 (37)	0.38
Phenotype score at final evaluation, pts	5.4 ± 1.9	5.4 ± 1.8	5.2 ± 2.0	0.53

Values are means ± SD or *n*/*N* (%). SCD, sudden cardiac death; VAs, ventricular arrhythmias; NA, not applicable; LVDD, left ventricular end‐diastolic dimension; LVDS, left ventricular end‐systolic dimension; LVEF, left ventricular ejection fraction; ICD, implantable cardioverter defibrillator.

### Phenotypes of family members of probands in Japanese ARVD/C cohort

Fifty‐eight relatives from 26 families were genotyped at a mean age of 40 ± 21 years. Six (10%), one (2%), and 44 (76%) relatives met the criteria for ARVD/C in the definite, borderline, and possible categories, respectively. Three individuals had experienced lethal VAs prior to genotyping, one had a history of unexplained syncope, and two had shown asymptomatic frequent premature ventricular contractions on Holter recordings. The remaining 52 relatives were genotyped for the purpose of family screening in the absence of any ARVD/C‐related symptoms.

### Genotypes of Japanese ARVD/C probands and family members

**Figure 2:**
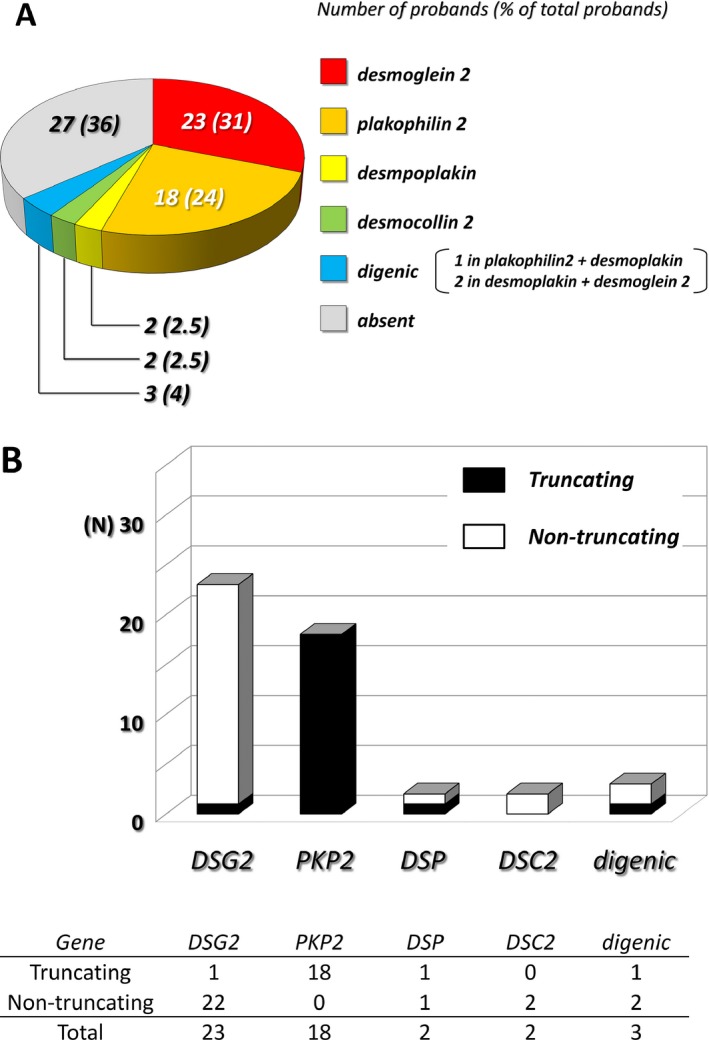
Genotype of Japanese ARVD/C probands. (A) Pie graph showing the number of probands who received the genetic test for ARVD/C. (B) Each bar graph was color‐coded according to the proportion of truncating or nontruncating mutations. Filled bar denotes truncating mutation, white bar denotes nontruncating mutation. Note that almost all *PKP2* mutations were truncating mutation, whereas the other mutations were almost nontruncating mutation.

Table 2 details identified mutations in bold, variants in plain italic, reference SNP ID, previous reports, MAF in Japanese controls, the number of carriers in the present cohort, and *in silico* prediction of protein changes. In 48 (64%) of the 75 probands, 30 pathogenic mutations were identified including seven premature stop‐gained (in seven probands), four frameshifts (in 11), two splice site mutations (in two), one multi‐exon deletion (in one) (Sonoda et al. 2017), and 16 missense mutations (in 30) (see details in Table 2 and Table S1). Of the 16 missense mutations, one (*DSG2*‐p.Arg46Trp) had previously been reported in a Caucasian ARVD/C cohort (Gehmlich et al. 2012; Groeneweg et al. 2015), nine had previously been reported by our group (Ohno et al. 2013), and six were novel. Of particular note, as described in Figure 2A, the most commonly affected genetic locus in Japanese ARVD/C probands was *DSG2* (10 mutations in 25 probands; monogenic in 23 probands and digenic in two probands) followed by *PKP2* (12 mutations in 19 probands; monogenic in 18 probands and digenic in one proband). Mutations in *DSP* (five mutations in five probands; monogenic in two probands and digenic in three probands) and *DSC2* (two double missense mutations in one and one frameshift in one) were far less common. Figure 2B demonstrates the contrasting distribution of missense and truncating mutation in *PKP2* and *DSG2*. Plakophilin 2 mutations were specifically dominated by truncating mutations, which accounted for 18 of 19 affected probands including digenic with desmoplakin mutation. Multiple mutations were identified mainly in *DSG2* as shown in Table S1.

**Table 2:** Desmosomal variants identified in Japanese ARVD/C cohort

Nucleotide change	Protein change (reference SNP ID)	Previous report	MAF in controls/covered alleles	Carriers in Probands (*N* = 75)	Carriers in relatives^b^ (*N* = 58)
Desmoglein 2					
c.1481 a>c	**p.Asp494Ala (rs193298428)**	Ohno et al. (2013)	0.006/2204	18 (4 homozygotes)	5
c.874 c>t	**p.Arg292Cys**	Novel	0.004/2212	14 (3 homozygotes)	10
c.136 c>t	**p.Arg46Trp**	Groeneweg et al. (2015)	Unreported	2	2
c.803 a>t	**p.Asn268Ile**	Novel	0.002/858	2	0
c.1562 a>g	**p.Asp521Gly**	Novel	0.002/2204	2	0
c.847g>a	**p.Glu283Lys**	Novel	Unreported	1	2
c.1448g>a	**p.Gly483Asp**	Novel	Unreported	1	0
c.1880‐1g>t	**Abnormal splice product**	Novel	Unreported	1	0
c.2681 t>g	**p.Leu894Trp**	Novel	0.001/742	1	0
c.1592 t>g	**p.Phe531Cys (rs200484060)**	Ohno et al. (2013)	0.002/858	1	1
*c.2536g>a*	*p.Asp846Asn*	Novel	Unreported	1	0
*c.2780 c>t*	*p.Pro927Leu* (rs146402368)	Ohno et al. (2013)	0.017/2292	2	0
*c.1597g>a*	*p.Val533Ile* (rs199761749)	Novel	0.005/2218	1	0
*c.3118g>a*	*p.Val1040Ile* (rs201966605)	Novel	Insufficient data	1	0
*c.1525g>a*	*p.Asp509Asn*	Novel	0.003/600	1	0
Plakophilin 2					
c.1725_1728 dupGATG	**p.Arg577fs**	Ohno et al. (2013)	Unreported	8	3
c.1132 c>t	p.Gln378*	Ohno et al. (2013); Groeneweg et al. (2015)	Unreported	1	0
c. 795‐811del	**p.Leu266fs**	Ohno et al. (2013)	Unreported	1	0
c.2119 c>t	p.Gln707*	Ohno et al. (2013)	Unreported	1	0
c.2095 c>t	p.Gln699*	Ohno et al. (2013)	Unreported	1	0
c. 1368‐1369insA	**p.Gln456fs**	Sonoda et al. (2017)	Unreported	1	0
c.2203 c>t	p.Arg735*	Novel^a^	Unreported	1	0
c.1035‐1g>a	**Abnormal splice product**	Novel	Unreported	1	0
Deletion exons 1‐14		Groeneweg et al. (2015); Sonoda et al. (2017)	Unreported	1	0
c.1951 c>t	p.Arg651*	Bao et al. (2013)	Unreported	1	0
c.1969g>t	p.Glu657*	Novel	Unreported	1	0
c.976g>a	**p.Ala326Thr (homozygote)** rs148480056	Ohno et al. (2013)	Unreported as homozygote	1	0
*c.2150 c>t*	*p.Pro717Leu* rs144018320	Ohno et al. (2013)	0.011/2202	6	0
*c.953A>C*	*p.His318Pro* rs181098323	Ohno et al. (2013)	Unreported	4	0
*39 a>g		Novel	Unreported	1	0
Desmoplakin					
c.8269g>c	**p.Asp2757His**	Ohno et al. (2013)	Insufficient data	1	0
c.4741 a>g	**p.Lys1581Glu (homozygote)** rs186842903	Ohno et al. (2013)	Unreported as homozygote	1	0
c.1203g>t	**p.Lys401Asn**	Ohno et al. (2013)	Unreported	1	1
c.593 a>c	**p.Gln198Pro (homozygote)**	Ohno et al. (2013)	Unreported as homozygote	1	0
c.4198 c>t	p.Arg1400*	Bao et al. (2013)	Unreported	1	2
*c.2360 a>g*	*p.Try787Cys*	Ohno et al. (2013)	0.005/1066	1	0
*c.8455 a>c*	*p.Met2819Leu* rs138329459	Ohno et al. (2013)	0.002/598	1	0
*c.6505 c>g*	*p.Gln2169Glu*	Novel	Unreported	1	0
Desmocollin 2					
c.394 c>t	**p.Arg132Cys**	Ohno et al. (2013)	Unreported	1	0
c.607 c>t	**p.Arg203Cys**	Ohno et al. (2013)	Unreported	1	1
c. 296_297 insA	**p.Ser99fs**	Novel	Unreported	1	0
*c.582 c>g*	*p.Asn194Lys*	Ohno et al. (2013)	Unreported	1	1

**Bold** denotes pathogenic mutation; *plain italic* denotes non‐pathogenic variant.

a Mutated mice were produced in Cardiovascular Development and Repair Department, Centro Nacional de Investigaciones Cardiovasculares, Madrid, Spain.

b Mutations or variants are heterozygous in relatives.

MAF, minor allele frequency.

Of the 58 family members, 21 had inherited pathogenic mutations, including two truncating mutations (either of which was found in five relatives). Table 2 listed mutations harbored among family members along with their proband relatives.

Regarding the most common variant p.Asp494Ala in *DSG2*, as shown in Table S2, linkage analysis revealed the fully same microsatellite pattern in one allele, and less divergent patterns in another allele among 18 probands with p.Asp494Ala in *DSG2*. In combination with the genealogical independency of probands, p.Asp494Ala variant in *DSG2* is thought for the first time to have a common founder.

### Outcomes of Japanese ARVD/C probands

During 6.4 ± 6.2 years of follow‐up after genotyping, recurrent lethal VAs occurred in 10 of the 43 probands who had histories of lethal VAs prior to genotyping, whereas *de novo* events developed in another 10 probands. A total of 53 (71%) probands exhibited lethal VAs at an average age of 45 ± 17 years. Five probands (6.4%) died during the follow‐up period, two from end‐stage heart failure (HF) at a mean age of 53 years and three from SCD at a mean age of 56 years. Lethal VAs occurred earlier in truncating mutation carriers than in either mutation negatives (35 ± 12 vs. 50 ± 19 years, *P* < 0.05) or nontruncating mutation carriers (35 ± 12 vs. 49 ± 16, *P* < 0.05).

**Figure 3:**
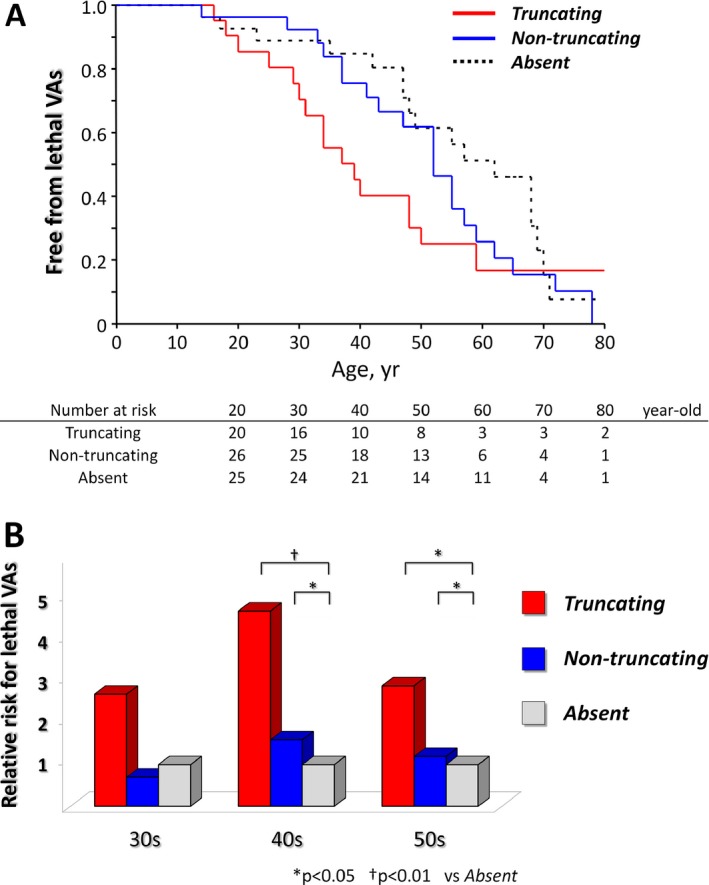
Survival curves and relative risk for lethal ventricular arrhythmias in probands. Kaplan–Meier survival curves for lethal ventricular arrhythmias (VAs) were constructed for groups sorted according to mutation status: (A) truncating mutation carriers, nontruncating mutation carriers, and mutation negatives. Relative risk was calculated according to age and mutation status (B) compared to mutation negatives (“absent”). Note the increased risk for lethal VAs by 40s among probands with truncating mutations.

Kaplan–Meier estimation for lethal VAs (Fig. 3A) did not show survival differences among each mutation status. Appropriate ICD or CRT‐D interventions for lethal VAs were administered in eight of 41 probands at an average of 5.8 ± 5.3 (0–13.3) years after ICD or CRT‐D implantation, mainly among candidates for secondary prevention (seven of eight probands). The appropriate ICD intervention rate during the mean follow‐up period of 4.85 years after implantation was 9.57%/year.

### Outcomes in family members of Japanese ARVD/C probands

Though probands had a higher incidence of life‐threatening events, their family members rarely developed ARVD/C‐related symptoms. Three relatives had experienced lethal VAs prior to genotyping; all these were nontruncating mutation carriers (*DSG2*‐R292C in two, and multiple mutations of *DSG2*‐R292C along with *DSG2*‐R46W in one). One relative with a truncating mutation (*PKP2*‐R577Dfs) developed *de novo* appropriate antitachycardia pacing therapy against VAs after prophylactic ICD implantation. The remaining 54 relatives remained free from VAs before and after genotyping.

### Age‐ and mutation‐dependent outcomes in Japanese ARVD/C probands

Because many probands, especially mutation positives, had early onset of manifestations, we grouped them into three categories according to time to onset: those exhibiting symptoms by their 30s (revealing risk at 29 years of age), those by their 40s (revealing risk at 39 years of age), and those by their 50s (revealing risk at 49 years of age). Figure 3B described the relative risks among different mutation status compared to mutation negatives. Cox proportional hazard analyses estimated that truncating mutation carriers had the highest arrhythmic risk, with 2.6‐(*P* = N.S.), 4.6‐(*P* < 0.01), and 2.9‐(*P* = 0.01) fold higher by their 30s, 40s, and 50s, respectively, compared to mutation negatives. Nontruncating mutation carriers showed similar trends in the age of onset with mutation negatives.

**Figure 4:**
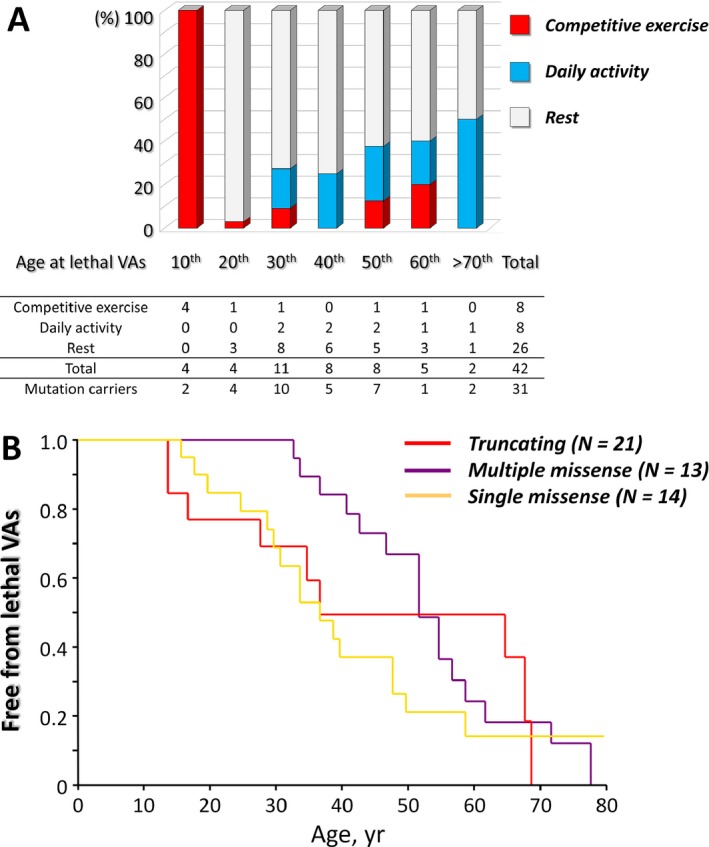
(A) Triggers of lethal arrhythmia in probands with lethal ventricular arrhythmias. Each bar is color‐coded according to the nature of the manifestation of lethal ventricular arrhythmias. (B) *Survivals associated with different types and numbers of mutations*. The missense mutation carriers shown in Figure 3A (blue line, nontruncating mutation carriers) were separated according to the number of mutation(s) harbored (yellow and purple line).

As the results above, age‐dependent difference in arrhythmic risk could be due to the acquisition over time of additional conditions other than mutation status such as physical stress. Although the incidence of lethal VAs was lower in the teen ages (<20 years), physical activity appeared most associated with occurrence of VAs (Fig. 4A). At variance with previous reports in Caucasians, survival analysis focusing on the number of mutations (Fig. 4B) showed no positive correlation between the number of mutations and adverse outcomes.

### Genotype–phenotype correlation in Japanese ARVC probands

Table 3 shows the clinical characteristics associated with different mutation statuses: (1) truncating and (2) nontruncating mutation carriers, and (3) mutation negatives. Repolarization anomaly was the most probable sign of mutation‐positive status (*P* < 0.01, Table 3). Reduced LVEF was more frequently observed in nontruncating mutation carriers than in truncating mutation carriers or mutation negatives (LVEF was reduced to 46%, 58%, and 55%, respectively). The same result was found in a different population of 88 probands selected based on “the modified TFC” (see details in Table S3). According to logistic regression analysis, as described in Figure S1, positive mutation status was closely associated with major repolarization anomaly (OR = 3.52, *P* = 0.005) and T‐wave inversion in inferior leads (OR = 3.18, *P* = 0.01). In particular, T‐wave inversion over V3‐4 strongly suggested that a proband was a mutation carrier (OR = 4.08, *P* = 0.002).

**Table 3:** Clinical characteristics associated with mutation status at genotyping

	Truncating mutation (+) *N* = 21	Nontruncating mutation (+) *N* = 27	Mutation negative *N* = 27	*P* value
Age at first evaluation, yrs	40 ± 18	43 ± 17	49 ± 18	0.16
Age at genotyping, yrs	44 ± 20	49 ± 16	52 ± 18	0.29
Age at final evaluation, yrs	46 ± 20	51 ± 16	54 ± 19	0.34
Follow‐up period, yrs	6.1 ± 6.9	8.0 ± 6.5^a^	4.6 ± 4.8^a^	0.08
Male, *n* (%)	16 (76)	19 (70)	20 (74)	0.71
First manifestation				
Lethal VAs, *n* (%)	14 (67)	15 (56)	14 (52)	N.S.^c^
Nonlethal VAs, *n* (%)	2 (10)	5 (19)	5 (19)	N.S.^c^
Heart failure, *n* (%)	2 (10)	2 (7)	1 (4)	N.S.^c^
Family history, *n* (%)	1 (5)	1 (4)	0	N.S.^c^
Syncope, *n* (%)	2 (10)	2 (7)	5 (19)	N.S.^c^
Others, (%)	0	1 (4)	2 (7)	N.S.^c^
Major structural anomaly, (%)	14 (67)	18 (67)	19 (70)	0.78
Major repolarization anomaly, *n* (%)	19 (90)^a^, ^b^	16 (59)^a^	12 (44)^b^	0.006
Major depolarization anomaly, *n* (%)	4 (19)^a^	13 (48)^a^	12 (44)^a^	0.09
Major ARVD/C‐related VAs, *n* (%)	2 (10)	8 (30)	8 (30)	0.18
Phenotype score, pts	5.0 ± 1.5	5.2 ± 1.8	5.3 ± 1.9	0.93
LVDD, mm	46 ± 7	49 ± 7	47 ± 6	0.12
LVDS, mm	31 ± 8^a^	35 ± 7^c^	33 ± 5^a^	0.03
LVEF, %	58 ± 14^a^	46 ± 15^a^	55 ± 12	0.03
ICD implantation at final evaluation, *n* (%)	9 (43)	16 (59)	16 (59)	0.42

Values are mean ± SD or *n*/*N* (%). Abbreviations as in Table 1.

a *P* < 0.05 between annotated values.

b *P* < 0.01 between annotated values.

c *P* = N.S. between each category.

**Figure 5:**
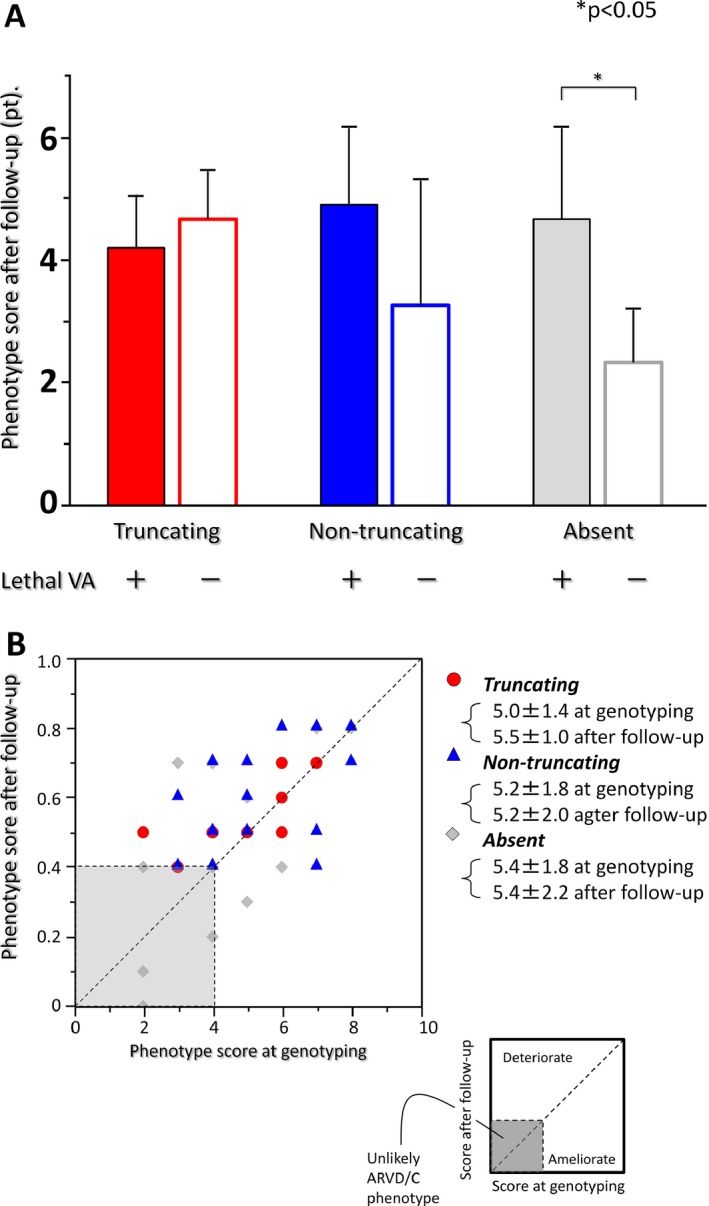
Phenotype scores associated with different mutation statuses. (A) Bar charts indicate the phenotype scores associated with different mutation statuses and outcomes. Note that higher phenotype score influenced outcome in nontruncating mutation carriers and mutation negatives, whereas phenotype severity was independent of outcome in truncating mutation carriers. Filled bars represent lethal ventricular arrhythmia victims, shaded bars represent intact individuals. (B) Phenotype alteration was depicted by plotting score at genotyping (*X*‐axis) and after follow‐up (*Y*‐axis). Value in each axis denotes phenotype score derived from “the modified 2010 TFC.”

Figure 5A shows phenotype scores calculated as described in the method section as bar graphs in three different mutation statuses. Among truncating mutation carriers (to the left two bars), phenotype scores were not different irrespective of VAs (5.4 vs. 6.0 points, respectively, *P* = N.S.). In contrast, among mutation negatives and nontruncating mutation carriers, VAs victims showed more severe phenotype scores (6.3 vs. 4.2 points, *P* = 0.07 among nontruncating mutation carriers; 6.0 vs. 3.2 points, *P* < 0.05 among mutation negatives).

### Phenotype alteration in probands during follow‐up

In Figure 5B, phenotype scores at the study entry are plotted against those at the time of final evaluation (mean follow‐up of 6.4 ± 6.2 years). Red circles indicate patients with truncating mutations, blue triangles those with nontruncating mutations, and gray diamonds those without mutations. Patients with <4 points, who are therefore less likely to have ARVD/C, are shown in the shaded area. Phenotype progression appeared to be common in each mutation status. To note, the normalization of phenotype scores was never seen in the truncating mutation carriers.

## Discussion

### Major findings from the Japanese ARVD/C cohort

This study first detailed the genetic background of non‐Caucasian ARVD/C probands and its impact on specific cardiac outcomes: (1) the genetic background of Japanese ARVD/C probands is distinct from that previously reported in Caucasian probands. *DSG2* mutations were predominant over those of *PKP2* or other desmosomal genes. (2) Cardiac outcome could be stratified by mutation status and age. (3) Phenotypic influence on lethal VAs was less potent in truncating mutation carriers whose arrhythmic risk was independent of phenotype severity.

### Racial disparity in genetic background between Caucasian and non‐Caucasian Japanese ARVD/C probands

In previous reports based on large numbers of Dutch and North‐American probands (Groeneweg et al. 2015), 63% of the Caucasian ARVD/C cohort have been mutation‐carriers. Asian probands have been uniformly scarce in these studies although more than 5% of U.S. citizens are of Asian descent (U. S. Census Bureau 2013). Our data revealed that the prevalence of desmosomal mutations in non‐Caucasian, clinically conclusive, Japanese ARVD/C probands is identical to that in Caucasians however; the most prevalent gene mutation was *DSG2*. We had not found this unique dominancy of *DSG2* in the previous report (Ohno et al. 2013) in which we had concluded *PKP2* as the most affected gene in 2013. This critical difference was brought by both the change in inclusion criteria and the increase in number of enrolls. As shown in the Table S3, mutation carriers are likely to show advanced phenotype, and conversely, the increase in number of probands leaded the increase in mutation carriers. We further employed more strict definition in mutational interpretation than before. Two variants in *PKP2* (*PKP2*‐P717L, *PKP2*‐H318P) as well as one variant in *DSG2* (*DSG2*‐P927L) had been treated as “mutation” in the previous but they were considered as nonpathogenic variants in the present research. Consequently, the increase in number of probands also leaded to the predominance of *DSG2* mutations.

Surprisingly, Chinese ARVD/C probands showed higher prevalence of *PKP2* mutations than other desmosomal mutations (Zhou et al. 2015), though not all subjects conclusively fulfilled the diagnostic criteria (Bao et al. 2013).

Notably, missense mutations in *DSG2* in our cohort as well as common polymorphisms in *DSG2* in Japanese controls were largely located in the extracellular domain (amino acid residues 1‐609). In contrast, missense mutations in *DSG2* in Caucasian probands are reportedly located in the extracellular region, but its polymorphisms are in the transmembrane and intracellular domain (amino acid residues 610‐1118), suggesting that only mutations in the extracellular component may be malignant (Kapplinger et al. 2011). But this seems not true in Japanese, and thus *DSG2* may be more vulnerable for the occurrence of *de novo* variants in Japanese.

### Genotype‐specific risk stratification

Previous genotype‐specific risk analyses among Caucasians have concluded that mutation status influences relatives more strongly than it does probands because even probands without mutations will develop symptoms in the near future. This finding implies that genotypic characterization may not be as helpful as it had been thought to be in predicting outcomes in Caucasian probands. In Japanese probands, event‐free survival is intergraded with age only among truncating mutation carriers. *PKP2*‐related early manifestation (Ohno et al. 2013) has still been constantly observed even though considerable numbers of *DSG2* mutation carriers were enrolled. Given that all lethal events among young victims in our cohort occurred during competitive exercise, exercise avoidance is the most effective especially among young carriers not only for preventing young carriers from disease development, but from fatal events. The follow‐up data of relatives showed very few adverse events though, all three relatives with lethal VAs (from different families) had desmosomal mutations; interestingly, all three mutations were nontruncating. The five relatives carrying truncating mutations, in contrast, did not conclusively fulfill the diagnostic criteria and did not experience adverse outcomes at the moment. Whether truncating mutation itself in relatives can affect either phenotype or outcome must be further investigated in non‐Caucasian populations.

### Influence of phenotype on outcome differs according to mutation status

Preceding reports have strongly suggested the extrinsic regulation of phenotype development (Nava et al. 2000; Pilichou et al. 2009; James et al. 2013). In this study, combined phenotype score did not differ between mutation positives and negatives, nevertheless, this report did clearly show the significant link between clinical outcomes and genetic backgrounds. Desmosomal mutations, especially truncating ones in probands, per se played a harmful role in outcome even when phenotype score was relatively low. Consequently, probands other than truncating mutation carriers could be further separated into high‐risk group with advanced phenotype and low‐risk group with mild phenotype.

### Limitations

First, this study cohort contained insufficient numbers of relatives with available clinical data, which prevented us from describing phenotype spectrum, survival, and disease course among family members. Second, phenotypes were identified mainly by ECG and structural findings, resulting in a lack of information about tissue characteristics because most of the tissue samples were obtained not from the right ventricle free wall but rather from the interventricular septum. Third, variant interpretation was based on a Japanese national database derived from exome sequencing of 1208 individuals at most and genotyping data from 3248 individuals; thus we could not completely dispel the possibility that some preclinical ARVD/C probands, especially those with missense variants, were incorporated among the controls.

## Conclusion

This survey revealed the divergent genetic and phenotypic characteristics of ARVD/C in a mono‐racial, non‐Caucasian population, leading to the mutation‐dependent prognosis which has never been concluded in Caucasian probands. Japanese probands are most affected by *DSG2* missense mutations followed by *PKP2* truncating mutations. Truncating mutations per se are responsible for the early onset of lethal VAs regardless of phenotype severity. Further investigation into the interaction between race and genetic background on disease penetrance and outcome is required.

## Disclosures

None.

## Sources of Funding

This work was supported by a Grant‐in‐Aid for Scientific Research from the Japan Society for the Promotion of Science (M.H., 15H04818, S.O., 15K09689d), the Japan Ministry of Health, Labour and Welfare (M.H., H24‐033, H26‐040, H27‐032), and Funds for Translational Research from the Japan Circulation Society (M.H.).

## Supporting information


**Appendix S1**. Method: Interpretation for missense variant.
**Table S1**. Details of multiple mutations in Table 2.
**Table S2**. Repeat numbers in a microsatellite 15xGT and an additional marker among 18 probands with p.Asp494Ala (*DSG2*‐D494A) variant.
**Table S3**. Clinical characteristics of 88 probands with more than “possible” criteria by “the modified TFC”.
**Figure S1**. Odds for mutation possession in 88 probands independent of genotypes.
