# Supplementary material for: Unique genetic background and outcome of non‐Caucasian Japanese probands with arrhythmogenic right ventricular dysplasia/cardiomyopathy
Source: Mol Genet Genomic Med. 2017 Aug 13;5(6):639–51. doi: 10.1002/mgg3.311 (PMC5702570; doi:10.1002/mgg3.311)
Supplement: Supplementary file 1 — Appendix S1. Method: Interpretation for missense variant. Table S1. Details of multiple mutations in Table 2. Table S2. Repeat numbers in a microsatellite 15xGT and an additional marker among 18 probands with p.Asp494Ala (DSG2‐D494A) variant. Table S3. Clinical characteristics of 88 probands with more than “possible” criteria by “the modified TFC”. Figure S1. Odds for mutation possession in 88 probands independent of genotypes. [file MGG3-5-639-s001.docx]

**SUPPLEMENTAL MATERIAL**

**Method. Interpretation for missense variant**

Missense variant is determined as pathogenic when both of the following criteria were met:

1. Minor allele frequency less than 0.005
   1. in Japanese controls according to the Human Genetic Variation Database (HGVD; http://www.genome.med.kyoto-u.ac.jp/SnpDB/) derived from exome sequencing of 1208 individuals at most and genotyping data from 3248 individuals.
   2. in the 1000 Genome Project (http://browser.1000genomes.org/index.html) if index variant was not listed in HGVD.
2. At least two out of three *in silico* prediction programs predicted the gene to be deleterious as below,
   1. SIFT score < 0.1 (http://sift.jcvi.org/)
   2. PolyPhen-II score > 0.90 (http://genetics.bwh.harvard.edu/pph2/)
   3. Combined Annotation Dependent Depletion (CADD) score > 20 (http://cadd.gs.washington.edu/)

**Table S1.** **Details of multiple mutations in Table 2.**

| Gene / amino acid change (1) | Gene / amino acid change (2) | Carriers in Proband  (N = 75) | Carriers in family members  (N = 58) |
| --- | --- | --- | --- |
| ***Digenic mutations*** | | | |
| Plakophilin2 / p.Ala326Thr (Homo) | Desmoplakin / p.Gln198Pro (Homo) | 1 | 0 |
| Desmoglein2 / p.Phe531Cys | Desmoplakin / p.Lys1581Glu (Homo) | 1 | 0 |
| Desmoglein2 / p.Arg292Cys | Desmoplakin / p.Arg1400* | 1 | 1 |
| ***Monogenic double mutation (cis-)*** | | | |
| Desmocollin2 / p.Arg132Cys | Desmocollin2 / p.Arg203Cys | 1 | 0 |
| ***Monogenic double mutation (trans-)*** | | | |
| Desmoglein2 / p.Arg292Cys | Desmoglein2 / p.Asp521Gly | 1 | 0 |
| ***Monogenic double mutation (undetermined location)*** | | | |
| Desmoglein2 / p.Arg292Cys | Desmoglein2 / p.Arg46Trp | 1 | 1 |
| Desmoglein2 / p.Gly483Asp | Desmoglein2 / p.Asp521Gly | 1 | 0 |
| ***Monogenic homozygous mutation*** | | | |
| Desmoglein2 / p.Asp494Ala | | 4 | 0 |
| Desmoglein2 / p.Arg292Cys | | 3 | 0 |

Abbreviations: Homo, homozygote.

**TableS2** Repeat numbers in a microsatellite 15xGT and an additional marker among 18 probands with p.Asp494Ala (*DSG2*-D494A) variant.

| Proband# | *DSG2* variant other than *DSG2*-D494A | 15xGT repeat numbers, allele1 | 15xGT repeat numbers, allele2 | T>C in the fifth GT |
| --- | --- | --- | --- | --- |
| D11 | *DSG2*-R46W | 13 | 13 | + (homo) |
| D1 | *DSG2*-D494A (homo) | 13 | 13 | + (homo) |
| D2 | *DSG2*-D494A (homo) | 13 | 13 | + (homo) |
| D12 | *DSG2*-R292C | 13 | 13 | + (homo) |
| D3 | *DSG2*-D494A (homo) | 13 | 13 | + (homo) |
| D13 | *DSG2*-R292C | 13 | 13 | + (homo) |
| D14 | *DSG2*-R292C | 13 | 13 | + (homo) |
| D5 | - (hetero) | 13 | 13 | + (homo) |
| D15 | *DSG2*-E283K | 13 | 16 | + (hetero) |
| D6 | - (hetero) | 13 | 16 | + (hetero) |
| D16 | *DSG2*-R292C | 13 | 13 | + (homo) |
| D17 | *DSG2*-R292C | 13 | 13 | + (homo) |
| D4 | *DSG2*-D494A (homo) | 13 | 13 | + (homo) |
| D7 | - (hetero) | 13 | 16 | + (hetero) |
| D8 | - (hetero) | 13 | 15 | + (hetero) |
| D18 | *DSG2*-N268I | 13 | 16 | + (hetero) |
| D9 | - (hetero) | 13 | 13 | + (homo) |
| D10 | - (hetero) | 13 | 13 | + (homo) |

**Table S3**. **Clinical characteristics of 88 probands with more than “possible” criteria by “the modified TFC”.**

|  | **Probands**  **N = 88** | **Mutation (+)**  **N = 51** | **Mutation (-)**  **N = 37** | **p value** |
| --- | --- | --- | --- | --- |
| Age at first evaluation, yr | 42±19 | 40±19 | 44±20 | 0.30 |
| Age at genotyping, yr | 46±20 | 45±19 | 47±21 | 0.60 |
| Age at final evaluation, yr | 48±20 | 47±19 | 48±22 | 0.72 |
| Follow-up period, yr | 6.0±5.9 | 7.1±6.6 | 4.6±4.7 | 0.05 |
| Male, n (%) | 62 (70) | 35 (69) | 27 (73) | 0.66 |
| First manifestation |  |  |  |  |
| Lethal VAs, n (%) | 53 (60) | 30 (58) | 23 (62) | 0.75 |
| Non-lethal VAs, n (%) | 14 (16) | 8 (16) | 6 (16) | 0.95 |
| Heart failure, n (%) | 5 (6) | 4 (8) | 1 (3) | 0.30 |
| Family history, n (%) | 2 (2) | 2 (4) | 0 | 0.22 |
| Syncope, n (%) | 9 (10) | 5 (10) | 4 (11) | 0.88 |
| Others, (%) | 4 (5) | 1 (2) | 3 (8) | 0.17 |
| Major structural abnormality, (%) | 54 (61) | 33 (65) | 21 (57) | 0.45 |
| Major repolarization abnormality, n (%) | 51 (58) | 36 (71) | 15 (41) | 0.005 |
| Major depolarization abnormality, n (%) | 32 (36) | 17 (33) | 15 (41) | 0.49 |
| Major ARVD/C-related VAs, n (%) | 23 (26) | 10 (20) | 13 (35) | 0.10 |
| Phenotype score at genotyping, pt | 4.9±1.8 | 5.2±1.7 | 4.5±1.9 | 0.06 |
| LVDD, mm | 47±7 | 47±8 | 46±6 | 0.64 |
| LVDS, mm | 32±7 | 33±8 | 32±5 | 0.93 |
| LVEF, % | 54±14 | 52±16 | 57±12 | 0.29 |
| ICD implantation at final evaluation, n (%) | 47 (55) | 25 (49) | 22 (65) | 0.15 |

Abbreviations:

NA, not applicable; VAs, ventricular arrhythmias; LVDD, left ventricular end-diastolic diameter; LVDS, left ventricular end-systolic diameter; LVEF, left ventricular ejection fraction; ICD, implantable cardioverter defibrillator.

**Figure S1**. **Odds for mutation possession in 88 probands independent of genotypes.**


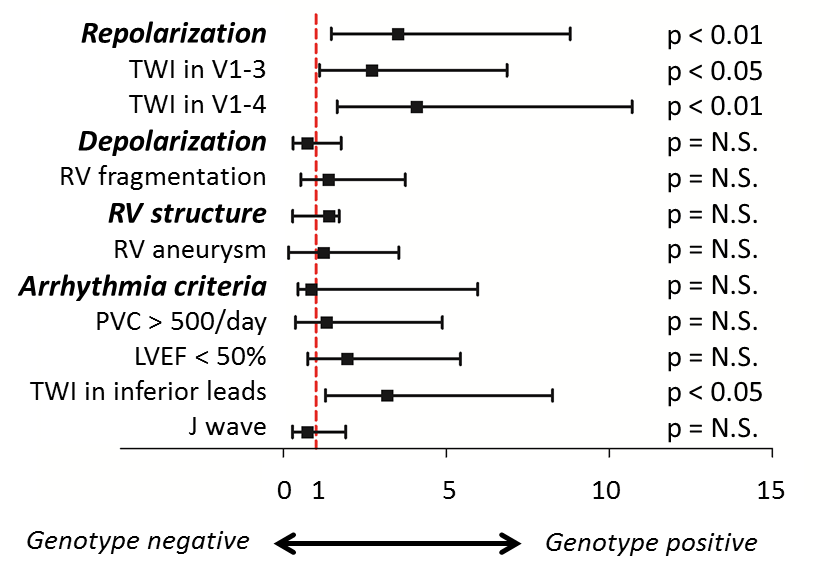


Abbreviations: Repolarization, major repolarization anomaly in the 2010TFC; TWI, T wave inversion; Depolarization, major depolarization anomaly in the 2010TFC; RV structure, major right ventricular structural anomaly in the 2010 TFC; Arrhythmia criteria, major arrhythmia in the 2010TFC; PVC, premature ventricular contraction.
